# Contact endoscopy as a novel technique for intra-operative identification of normal pituitary gland and adenoma

**DOI:** 10.3171/2021.10.FOCVID21199

**Published:** 2022-01-01

**Authors:** Christina Jackson, Derek Kai Kong, Zachary C. Gersey, Eric W. Wang, Georgios Zenonos, Carl H. Snyderman, Paul A. Gardner

**Affiliations:** Departments of 1 Neurological Surgery and; 2 Otolaryngology, University of Pittsburgh Medical Center, Pittsburgh, Pennsylvania

**Keywords:** endoscopic endonasal approach, contact endoscopy, intraoperative visualization, pituitary adenoma

## Abstract

Intraoperative distinction of pituitary adenoma from normal gland is critical in maximizing tumor resection without compromising pituitary function. Contact endoscopy provides a noninvasive technique that allows for real-time in vivo visualization of differences in tissue vascularity. Two illustrative cases of endoscopic endonasal approaches (EEAs) for resection of pituitary adenoma illustrate the use of contact endoscopy in identifying tumor from gland and differentiating a thin section of normal gland draped over the underlying tumor, thereby allowing for safe extracapsular tumor resection. Contact endoscopy may be used as an adjunct for intraoperative, in vivo differentiation of pituitary gland and adenoma.

The video can be found here: https://stream.cadmore.media/r10.3171/2021.10.FOCVID21199

## Transcript

This is a video demonstrating a new concept using contact endoscopy to try to differentiate between normal pituitary gland and adenoma during endoscopic surgery.

### 0:33 Normal Pituitary Gland Vasculature.

A normal pituitary gland typically demonstrates very classic microvascular pattern we can sometimes differentiate.^1,2^

### 0:42 Enhanced Endoscopy.

This kind of differentiation of vascularity has been used successfully in contact endoscopy in head and neck surgery, especially for laryngeal surgery.^3–6^

### 0:50 Storz Professional Imaging Enhancement System.

We took the same endoscope, which in this case was produced by the KARL STORZ company, and used it to try to look at pituitary tumors and adenoma.^5^

### 1:02 Enhanced Endoscopy in EEA.

Here is an example of using the contact endoscopy. You can see the type of microscopic view essentially that we can get by placing the contact endoscope very close or on the tissue. Here you can see a microvascular pattern, and as we come in contact and adjust the focus, we can start to see that microvascular pattern come into closer view. This does require a certain learning curve with understanding using of the scope. We can really pick out that microvascular pattern that may not be evident and usually is not so evident under simple white light endoscopy. Here is a beautiful example of normal gland with a really very hypervascular pattern to it. And using this over several cases, we started to gain an appreciation for the differentiation between normal gland and tumor.

### 1:57 Contact Endoscopy of Adenoma and Gland.

Here is an example where we are looking at both. You can see the endoscopy of the tumor on the left and clearly has almost no vascularity to it. You can see a few random blood cells. Whereas on the right, the gland has a very typical microvascular pattern with a very clear archetypal network to it. Here is that visualization, and again using this to differentiate between tumor and gland. Here is yet another example of a different tumor. Again, you can see the lack of vascularity in the tumor, and once we focus on the gland tissue itself, you can really see a bit of that microvascular pattern.

### 2:42 Illustrative Case 1.

This case demonstrates a 68-year-old man who presented with more than a year of double vision and also decreased libido. Neurological exam showed a complete sixth nerve palsy. We find a large sellar and suprasellar mass with clear extension into the left cavernous sinus. He has some evidence of mild pituitary dysfunction.

### 3:07 ICG Fluorescence.

Intraoperatively after wide exposure, with indocyanine green fluorescence, we can certainly see vasculature well and the edge of the tumor shows appropriate bleeding from the gland.

### 3:18 Contact Endoscopy on Tumor.

When we exposed the tumor itself and performed contact endoscopy, we see what seems to be a relatively common pattern, which is this very bland architecture in the tumor. We don’t see any clear vascular pattern. We don’t see a network of vasculature. We can certainly see small areas of capillaries around the edge and bleeding from the edge, but no frank visible network. There is some learning curve with using the contact endoscope to appropriately focus it within the tumor itself. But rather quickly, we were able to do this reliably on every case.

### 4:00 Tumor Removal.

We then proceeded with resection in the standard fashion. We sent some specimen for pathology and then, using two suctions, resecting the tumor back to the posterior aspect of the sella.

### 4:11 Left Cavernous Sinus.

And then visualize the left cavernous wall, which clearly had invasion through it. And here we can see after filling the cavernous sinus with SURGIFLO, Floseal, and then working up to the right cavernous wall.

### 4:27 Right Medial Cavernous Wall.

The right cavernous wall here is not invaded; in fact, there is evidence of a small amount of gland on that right cavernous wall. This is a very clear example of the medial cavernous wall in this case on both sides. Clearing first the left and now the right side, then that allows us to finally peel the tumor from the diaphragma. Again, some specimen being grasped here with a pituitary, and very gentle countertraction and dissection can even be done with the suction. Care is taken to not just pull the tumor out, and here we sharply dissect the gland pseudocapsule from the tumor.

### 5:04 Resection of Left Cavernous Wall.

Here we can see very clear separation of the medial cavernous wall and then resection by cutting the caroticoclinoidal ligament as the last cut to perform our final resection that has invaded the medial cavernous wall. The inferior aspect of it is probably the most invaded portion here as we cut the inferior parasellar ligament. And finally remove the medial cavernous wall.

### 5:25

Angled endoscopy is used to inspect the area between the diaphragma and the anterior dura. Again, we can clearly see a classic pattern with visualization of the gland along the diaphragma.

### 5:45 Contact Endoscopy on Gland.

We completely resected the cavernous wall, and then contact endoscopy on the gland shows what seems to be a typical pattern of the vascular network, which shows the architecture of the gland itself and helps us identify and separate the gland from the tumor itself.

No evidence of any damage with the contact endoscopy with any heat transmission. Of note, we do use an LED light source at this point, which is not quite at full power, but you can see the visualization is really quite good. You can even see red blood cells flowing through the capillaries in this vascular network on the gland.

### 6:17

Tissue without this vascular network which was felt to be tumor was sent for pathology, and histology was confirmed to be adenoma without normal pituitary architecture.

### 6:27 Illustrative Case 2.

Another case, 75-year-old man who presented, you can see notable labs has some stalk effect, hyperprolactinemia.

### 6:37

Indocyanine green fluorescence does not really differentiate in some cases, and it really has a narrow window. Here we see an area where we think might be a layer of gland overlying the tumor. But as is always the case, it can be very difficult to differentiate the two simply through white light.

### 6:54 Contact Endoscopy on Tumor.

By adding contact endoscopy to view the tumor, here we can clearly see lack of any vascular pattern, any vascular network. We can see very low vascularity, which is very typical for tumor in this case. It does have some vessels in it or red blood cells in it, but no organized network. This again differentiates between gland.

### 7:20 Contact Endoscopy on Lip of Gland.

And where we do contact endoscopy here on the gland, and you can see the network and the architecture of the gland itself. And here we can see that vasculature. Even though this is a very thin layer, that thin layer still had its vasculature and its architecture preserved. So this allows us in situ, in vivo to evaluate this without having to cut out any of the tissue, send it to pathology, or cut it to see whether or not it bleeds, which is another technique that can be used.

### 7:46 Extracapsular Dissection.

And in this case, then we can be confident of this capsule and able to perform an extracapsular dissection of the tumor by peeling it free from the area that we are now convinced was gland. This is typical after extracapsular dissection; we can then peel the tumor out, and here you can see where that pseudocapsule, which remember is comprised of pituicytes, has been separated from the tumor. And here is our final view, with now very obvious gland now preserved on the surface.

### 8:13

Again, this tissue, which was felt to be tumor without any vascular pattern to it, was sent for histopathology and was confirmed to be adenoma.

### 8:22 Conclusions.

Obviously, further study is necessary to confirm or differentiate between these areas and ensure what we are seeing with contact endoscopy always fits the histology, which remains to be determined in a large series. However, I think this is an exciting technique that can be used to help preserve gland and help ensure the most complete resection of pituitary tumors. This allows again for real-time assessment in situ of the architecture of pituitary gland. It has this great advantage of examining multiple areas without having to cut into the tissue itself. It requires further study, but we are excited to look into this further. Thank you very much.

## Disclosures

Drs. Snyderman and Gardner reported “other” from SPIWay, LLC during the conduct of the study.

## Author Contributions

Primary surgeon: Gardner, Snyderman. Editing and drafting the video and abstract: Gardner, Jackson, Kong, Gersey, Zenonos. Critically revising the work: all authors. Reviewed submitted version of the work: Gardner, Jackson, Kong, Wang, Gersey, Zenonos. Approved the final version of the work on behalf of all authors: Gardner. Supervision: Gardner, Wang, Snyderman.

## Supplemental Information

### Patient Informed Consent

Informed consent was obtained under IRB no. STUDY20070049. The patient provided consent to the submission and publication of the related surgical video.
